# Lymphovascular invasion status at transurethral resection of bladder tumors may predict subsequent poor response of T1 tumors to bacillus Calmette-Guérin

**DOI:** 10.1186/s12894-016-0122-1

**Published:** 2016-01-19

**Authors:** Keishiro Fukumoto, Eiji Kikuchi, Shuji Mikami, Akira Miyajima, Mototsugu Oya

**Affiliations:** Department of Urology, Keio University School of Medicine, 35 Shinanomachi, Shinjuku-ku, Tokyo, 160-0016 Japan; Division of Diagnostic Pathology, Keio University School of Medicine, Tokyo, Japan

**Keywords:** Urinary bladder neoplasms, Carcinoma, Transitional cell, Lymphatic metastasis, Recurrence, Disease progression

## Abstract

**Background:**

Lymphovascular invasion (LVI) is an important step in the process of tumor dissemination and metastasis outside the primary organ, but the relationship between LVI and the prognosis of T1 non-muscle invasive bladder cancer (NMIBC) has not been fully evaluated. Accordingly, the present study was performed to evaluate whether LVI had an impact on the clinical outcome in patients with T1 NMIBC.

**Methods:**

A total of 116 consecutive patients were diagnosed with T1 NMIBC from 1994 to 2013 at Keio University Hospital. All cases were reviewed by a single uro-pathologist. The prognostic significance of LVI was assessed in relation to recurrence and stage progression.

**Results:**

The median follow-up period was 53 months. LVI was histologically confirmed in 30 patients (25.9%). There were no significant differences of clinical features between the patients with and without LVI. In T1 patients, univariate analysis demonstrated that LVI positivity was associated with stage progression (*p* = 0.003), but not with tumor recurrence (*p* = 0.192). Multivariate analysis confirmed that LVI was independently associated with stage progression (*p* = 0.006, hazard ratio = 4.00). In 85 patients who received BCG instillation, LVI was independently associated with both tumor recurrence and stage progression (*p* = 0.036 and 0.024, hazard ratio = 2.19 and 3.76).

**Conclusions:**

LVI is a strong indicator of an increased risk of recurrence and progression in BCG-treated patients with T1 NMIBC. This information might assist clinicians to develop appropriate management and counseling strategies for these patients.

## Background

The optimum management and therapeutic strategy for T1 non-muscle invasive bladder cancer (NMIBC) are still being debated. As T1 NMIBC has a higher risk of recurrence and higher progression rate, current guidelines recommend adjuvant therapy with bacillus Calmette-Guérin (BCG) after transurethral resection of bladder tumor (TURBT) [1]. However, recurrence affects about half of all patients with T1 NMIBC who receive BCG therapy and 17 %–23 % show progression to muscle invasive tumors [2, 3]. Patients with a very high risk of recurrence and stage progression should receive more aggressive therapy such as immediate total cystectomy [4]. The clinical outcome of immediate cystectomy for T1 NMIBC is good, with the 10-year disease-specific survival rate being approximately 80 % [5, 6], but not all T1 patients need radical surgery, which has a relatively high morbidity rate and reduces the quality of life [7]. One of the major issues regarding management of T1 NMIBC is the lack of appropriate tools for identifying patients with a very high risk of stage progression. Various prognostic factors that predict a poor outcome of Ta/T1 NMIBC have been reported, including the sex [3], age [8, 9], tumor diameter [10], multifocality [8, 10], concomitant carcinoma in situ (CIS) [10, 11], CIS in the prostatic urethra [3], histological grade [8], and molecular grade determined by fluorescence in situ hybridization [12]. Lymphovascular invasion (LVI) is considered to be the most important step in the initiation of tumor dissemination/metastasis, and it has been identified as a strong indicator of a poor prognosis for various cancers, including carcinoma of the lung [13], breast [14], colon [15], kidney [16], and prostate [17], as well as urothelial carcinoma of the upper urinary tract [18, 19]. In patients with clinical stage I nonseminomatous testicular tumors, information on LVI status was reported to be important for making a decision about whether or not to provide adjuvant chemotherapy [20]. In patients with muscle invasive bladder cancer treated by total cystectomy, LVI is a strong indicator of poor survival, since the 10-year cancer-specific survival rate is 31.0 %–39.3 % for LVI-positive patients versus 72.0 %–73.6 % for LVI-negative patients [21, 22]. A few investigators have evaluated the influence of LVI in TURBT specimens of NMIBC patients, but conflicting results have been reported [23–26]. Therefore, we investigated the association of LVI status reviewed by a dedicated uro-pathologist with clinical background factors in T1 NMIBC patients and evaluated whether LVI was useful for identifying a higher risk of stage progression.

## Methods

### Patients

**Fig. 1:**
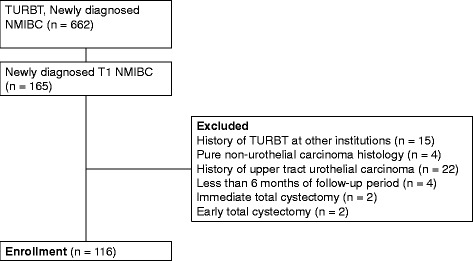
Flow diagram of the study population

A total of 662 patients with newly diagnosed NMIBC were treated from January 1994 to December 2013 at Keio University Hospital and 165 patients had T1 NMIBC (Fig. 1). Fifteen patients who received TURBT at other institutions were excluded. Patients with pure non-urothelial carcinoma (e.g., squamous cell carcinoma or adenocarcinoma), a history of upper tract urothelial carcinoma, and follow-up for less than 6 months were also excluded (4, 22, and 4 patients, respectively). Furthermore, we excluded 4 patients who had undergone total cystectomy without confirmation of progression. Two of them had received immediate total cystectomy, which meant that it was performed soon after TURBT without further intravesical therapy. The other 2 patients had received early total cystectomy, which was performed after recurrence but before stage progression. Finally, 116 subjects were included in this analysis, among whom 85 had received instillation of BCG after TURBT.

### Evaluation of resected specimens

**Fig. 2:**
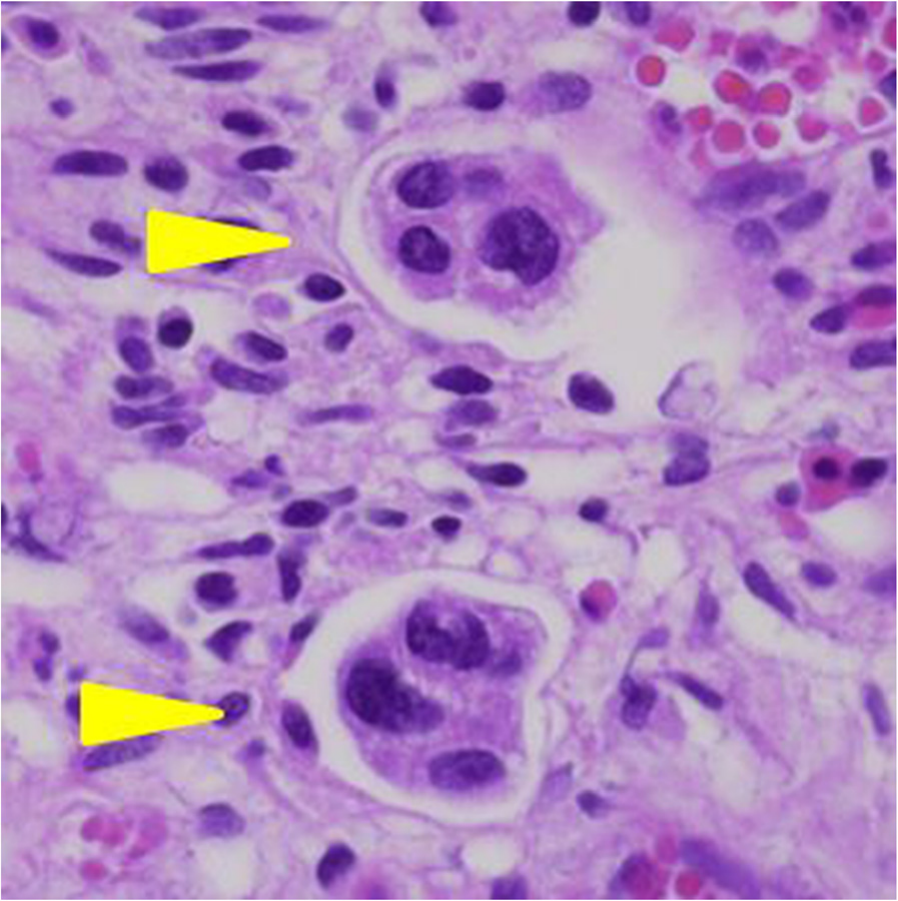
LVI (arrowhead) in a tumor specimen. Hematoxylin and eosin staining, × 400

All specimens were reviewed by a dedicated uro-pathologist who was unaware of the clinical outcome. Based on examination of hematoxylin and eosin stained sections, LVI was considered to be present when tumor cells were unequivocally noted within or attached to the walls of a vascular or lymphatic space (Fig. 2). Multiple serial sections were viewed and immunohistochemical markers for lymphatic channels (D2-40) and endothelial cells (CD31/34) were used in equivocal cases.

### Treatment

Intravesical BCG therapy was generally performed for intermediate or high risk NMIBC according to the current clinical guidelines [1]. However, the attending physician and/or patient sometimes decided against treatment with BCG because of side effects. Instillation of BCG was begun 4 to 5 weeks after TURBT and was continued at weekly intervals for 6 to 8 weeks at a dose of 80 mg (Tokyo 172 strain) or 81 mg (Connaught strain). Patients were followed postoperatively with cystoscopy and urinary cytology every 3 months for 2 years, every 6 months for the next 3 years, and annually thereafter. Excretory urography and/or computed tomography were performed to evaluate the upper urinary tract every year for 5 years after treatment.

### Endpoints

We defined tumor recurrence as any evidence of disease on follow-up evaluation, while progression was defined as muscle invasion or metastasis. Recurrence-free survival time was calculated as the interval between TURBT and the date of tumor recurrence, while progression-free survival was determined from the date of TURBT to progression. Cancer-specific survival and overall survival were based on death from bladder cancer and death from any cause, respectively.

### Statistical analysis

Variables were compared between different groups by using the χ2 test. Recurrence-and progression-free survival rates were estimated by the Kaplan-Meier method. Survival curves were compared with the log-rank test. Univariate and multivariate analyses of tumor recurrence and stage progression were done using the Cox proportional hazards model with stepwise forward regression. The independent variables included in survival analysis were patient age (<70 vs. ≥70 years), sex, tumor grade (G1/2 vs. G3), concomitant CIS, multifocality, intravesical BCG therapy, intravesical chemotherapy, a history of Ta NMIBC, and LVI status (positive or negative). Differences between groups were regarded as significant at *P* < 0.05. All analyses were performed with the SPSS v. 21.0 statistical software package (IBM Corp., Somers, NY).

### Ethics and consent

This study was conducted subject to the guidelines of the Declaration of Helsinki and approved by our ethical committee. The reference number is 20130101. The ethical committee exempted obtaining informed consent because our study design was done by a retrospective fashion. Data were obtained from medical chart and patient identifying information was anonymized before analysis.

## Results

### Clinicopathological characteristics of the 116 patients

The median age of the patients was 70.6 years (range: 40 to 89 years). Men accounted for 84.5% of the patients (*n* = 98) and women for 15.5% (*n* = 18). LVI was histologically confirmed in 30 patients (25.9%). Table 1 presents the association between clinicopathological characteristics and LVI status in the 116 patients. There were no significant differences of clinical features between the LVI-positive and LVI-negative patients. During the median follow-up period of 53 months (range: 6–239 months), 47 of 116 patients (40.5%) experienced recurrence and 16 patients (13.8%) showed stage progression. Of the 16 patients with stage progressions, one had distant metastasis. Fourteen patients died (12.1%) and 7 patients (6.0%) died of their disease.

**Table 1:** Clinicopathological characteristics of 116 patients stratified according to LVI status

Characteristic	Total		LVI positive		LVI negative		*p* value
No. of patients	116		30		86		
Age							0.073
<70 years	55	(47.4 %)	10	(33.3 %)	45	(52.3 %)	
≥70 years	61	(52.6 %)	20	(66.7 %)	41	(47.7 %)	
Sex							0.256
Male	98	(84.5 %)	27	(90.0 %)	71	(82.6 %)	
Female	18	(15.5 %)	3	(10.0 %)	15	(17.4 %)	
Tumor grade							0.115
G1/2	7	(6.0 %)	0	(0.0%)	7	(8.1 %)	
G3	109	(94.0 %)	30	(100.0%)	79	(91.9 %)	
Concomitant CIS							0.247
Positive	26	(22.4 %)	9	(30.0 %)	17	(19.8 %)	
Negative	90	(77.6 %)	21	(70.0 %)	69	(80.2 %)	
Multifocality							0.638
Multiple	85	(73.3 %)	21	(70.0 %)	64	(74.4 %)	
Solitary	31	(26.7 %)	9	(30.0 %)	22	(25.6 %)	
BCG instillation							0.334
Yes	85	(73.3 %)	24	(80.0 %)	61	(70.9 %)	
No	31	(26.7 %)	6	(20.0 %)	25	(29.1 %)	
Intravesical chemotherapy							0.601
Yes	16	(13.8 %)	4	(13.3 %)	12	(14.0 %)	
No	100	(86.2 %)	26	(86.7%)	74	(86.0 %)	
History of Ta NMIBC							0.418
Recurrence	7	(6.0 %)	1	(3.3 %)	6	(7.0 %)	
Primary	109	(94.0 %)	29	(96.7 %)	80	(93.0 %)	

*LVI* Lymphovascular invasion, *CIS* Carcinoma in situ, *BCG* Bacillus Calmette-Guérin, *NMIBC* Non-muscle invasive bladder cancer

### Predictors of recurrence and stage progression in all patients

Univariate and multivariate analyses were performed to determine the predictors of tumor recurrence and stage progression (Table 2). Recurrence was noted in 16 patients (53.3%) from the LVI-positive group and 31 patients (36.0%) from the LVI-negative group. Treatment with BCG (*p* = 0.005) and intravesical chemotherapy (*p* = 0.007) had a significant influence on tumor recurrence according to univariate analysis. Multivariate Cox regression analysis showed that BCG therapy was an independent determinant of a lower risk of tumor recurrence (*p* = 0.007, hazard ratio (HR) = 0.44).

**Table 2:** Results of univariate and multivariate analyses

Characteristic	Recurrence-free survival			Progression-free survival		
	Univariate	Multivariate		Univariate	Multivariate	
	*p* value	HR (95 % CI)	*p* value	*p* value	HR (95 % CI)	*p* value
Age	0.133			0.775		
<70 years						
≥70 years						
Sex	0.166			0.861		
Male						
Female						
Tumor grade	0.916			0.289		
G1/2						
G3						
Concomitant CIS	0.653			0.925		
Positive						
Negative						
Multifocality	0.063			0.344		
Single						
Multiple						
BCG instillation	0.005		0.007	0.953		
Yes		0.44 (0.24–0.80)				
No						
Intravesical chemotherapy	0.007			0.631		
Yes						
No						
History of Ta NMIBC	0.735			0.245		
Recurrence						
Primary						
Lymphovascular invasion	0.192			0.003		0.006
Positive					4.00 (1.49–10.75)	
Negative						

Nine patients (30.0%) with LVI demonstrated stage progression, as did 7 patients (8.1%) without LVI. Kaplan-Meier analysis showed that patients in the LVI-positive group had a higher risk of stage progression, with the 5-year progression-free survival rate being 61.8% in LVI-positive patients and 90.4% in LVI-negative patients (*p* = 0.003). Multivariate analysis demonstrated that LVI had an independent influence on progression-free survival (*p* = 0.006, HR = 4.00).

### Predictors of recurrence and stage progression in patients treated with BCG

**Fig. 3:**
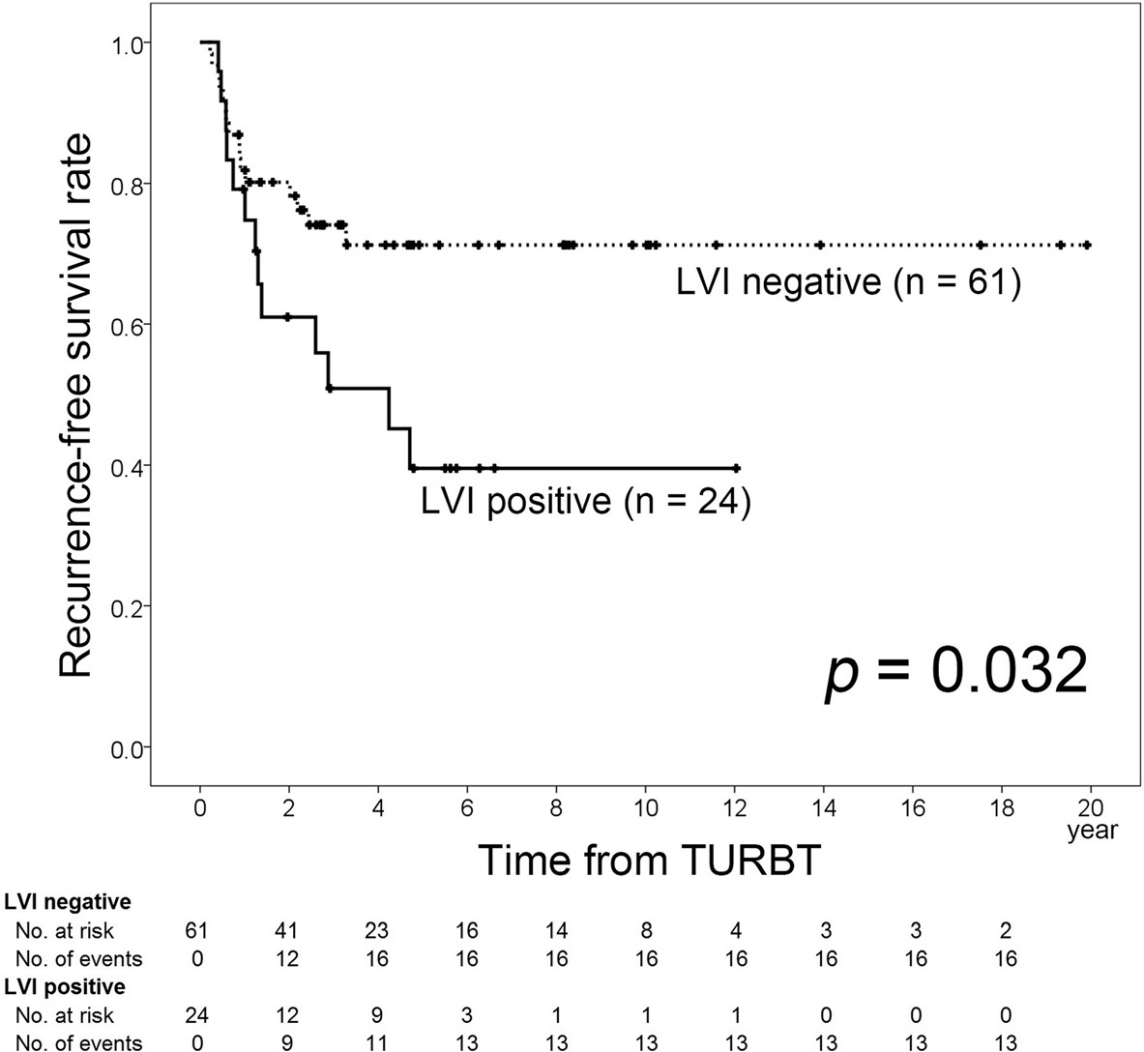
Recurrence-free survival rate according to LVI status in patients treated with BCG

**Fig. 4:**
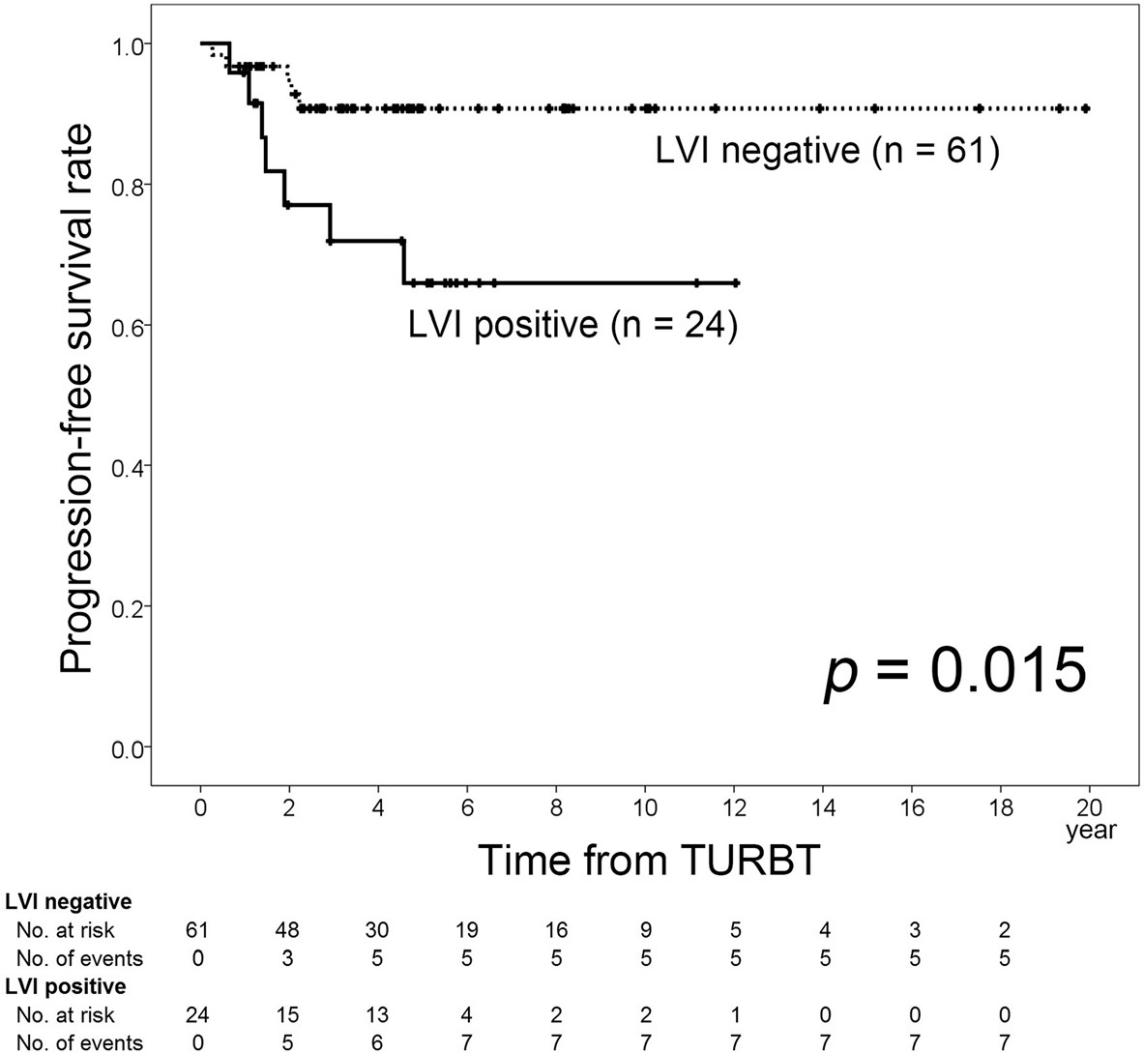
Progression-free survival rate according to LVI status in patients treated with BCG

We performed a subgroup analysis of the 85 patients who received BCG therapy. Their clinicopathological characteristics are listed in Table 3. There were no significant differences of clinical features between the LVI-positive and LVI-negative patients. We investigated whether LVI had a prognostic impact on tumor recurrence and stage progression (Table 4). Among the 85 patients, LVI was confirmed in 24 patients (28.2%). In the LVI-positive group, 13 patients (54.2%) experienced recurrence and 7 patients (29.2%) showed stage progression, while the corresponding numbers in the LVI-negative group were 16 (26.2%) and 5 (8.2%), respectively. Kaplan-Meier analysis revealed that the 5-year recurrence-free and progression-free survival rates of LVI-positive patients were 39.5% and 65.9%, respectively, which were significantly lower than those of LVI-negative patients (71.2% and 90.8%, *p* = 0.032 and 0.015, respectively; Figs. 3 and 4). Multivariate analysis confirmed that LVI had an independent influence on recurrence-free and progression-free survival in T1 NMIBC patients treated with BCG (*p* = 0.036 and 0.024, HR = 2.19 and 3.76, respectively).

**Table 3:** Clinicopathological characteristics of 85 BCG-treated patients stratified according to LVI status

Characteristic	Total		LVI positive		LVI negative		*P* value
No. of patients	85		24		61		
Age							0.074
<70 years	45	(52.9%)	9	(37.5%)	36	(59.0%)	
≥70 years	40	(47.1%)	15	(62.5%)	25	(41.0%)	
Sex							0.174
Male	71	(83.5%)	22	(91.7%)	49	(80.3%)	
Female	14	(16.5%)	2	(8.3%)	12	(19.7%)	
Tumor grade							0.258
G1/2	4	(4.7%)	0	(0.0%)	4	(6.6%)	
G3	81	(95.3%)	24	(100.0%)	57	(93.4%)	
Concomitant CIS							0.969
Positive	21	(24.7%)	6	(25.0%)	15	(24.6%)	
Negative	64	(75.3%)	18	(75.0%)	46	(75.4%)	
Multifocality							0.905
Multiple	61	(71.8%)	17	(70.8%)	44	(72.1%)	
Solitary	24	(28.2%)	7	(29.2%)	17	(27.9%)	
History of Ta NMIBC							0.563
Recurrence	5	(5.9%)	1	(4.2%)	4	(6.6%)	
Primary	80	(94.1%)	23	(95.8%)	57	(93.4%)

**Table 4:** Results of univariate and multivariate analyses in patients treated with BCG after TURBT

Characteristic	Recurrence-free survival			Progression-free survival		
	Univariate	Multivariate		Univariate	Multivariate	
	*p* value	HR (95% CI)	*p* value	*p* value	HR (95% CI)	*p* value
Age	0.095			0.523		
<70 years						
≥70 years						
Sex	0.677			0.491		
Male						
Female						
Tumor grade	0.593			0.424		
G1/2						
G3						
Concomitant CIS	0.728			0.943		
Positive						
Negative						
Multifocality	0.064			0.250		
Single						
Multiple						
History of Ta NMIBC	0.694			0.079		
Recurrence						
Primary						
Lymphovascular invasion	0.032		0.036	0.015		0.024
Positive		2.19 (1.05–4.55)			3.76 (1.19–11.90)	
Negative

### Association of LVI status with cancer-specific survival and overall survival

Among 16 patients with stage progression, 7 underwent total cystectomy, 3 were treated with radiation therapy, and 1 received systemic chemotherapy. Five patients received no treatment at their request. The 5-year cancer-specific survival rate of LVI-negative patients was 96.3%, which was marginally higher than that of LVI-positive patients (79.8%, *p* = 0.07). The 5-year overall survival rate of LVI-negative patients was 89.4%, which was not significantly different from that of LVI-positive patients (71.8%, *p* = 0.185). In the patients treated with BCG, LVI status was not associated with either cancer-specific survival or overall survival (*p* = 0.143 and 0.235, respectively).

## Discussion

Our study of 116 patients with T1 NMIBC revealed that LVI was significantly associated with stage progression after TURBT. In addition, we confirmed that LVI positivity was an independent risk factor for tumor recurrence and progression in T1 NMIBC patients treated with intravesical BCG instillation. To the best of our knowledge, this is the first report that LVI in TURBT specimens is significantly associated with recurrence and stage progression of T1 NMIBC after treatment with BCG.

T1 NMIBC has a high potential for recurrence, and some patients experience stage progression that requires more aggressive therapy such as total cystectomy. Therefore, it is critical to identify the subset of T1 NMIBC patients whose tumors are highly malignant and have the potential to progress to muscle invasive disease. Various biological markers (such as p16, pRb, p53, MIB-1, and HSP90) have been studied for identifying aggressive T1 tumors [27–29], but these are relatively expensive and require additional procedures for histopathological analysis. In contrast, LVI can easily be investigated during standard histopathological evaluation.

Several investigators have already investigated whether the LVI status of TURBT specimens was a prognostic factor in patients with NMIBC. Lopez et al. [23] reported an LVI positivity rate of 10% (17/170 T1 NMIBC patients) in their series and found that the LVI status was associated with overall survival. However, their patients were collected from 1983 to 1990 and were treated by adjuvant intravesical chemotherapy (mitomycin C or Adriamycin). Also, they did not determine the predictive value of LVI status for recurrence or stage progression by multivariate analysis. Andius et al. [24] analyzed 121 patients with T1 NMIBC, and found an association between LVI status and stage progression as well as cancer-specific survival. The same pathologist reviewed all histopathological material, as was done in our study, and LVI was confirmed in 12 patients (10%). In multivariate analysis, LVI was associated with both stage progression and cancer-specific survival. However, TURBT was performed between 1987 and 1988 in their study, and only one patient received adjuvant BCG instillation. Conflicting results with regard to the predictive value of LVI status for a poor clinical outcome have been reported in the era when BCG was established as standard adjuvant therapy for T1 NMIBC. Cho et al. [25] reviewed 118 patients with T1 NMIBC who underwent TURBT between 2001 and 2007, and evaluated the impact of LVI on tumor recurrence, stage progression, and metastasis. Two independent uro-pathologists reviewed the slides and found 33 LVI-positive patients (28%). Their multivariate analysis showed that LVI was significantly associated with tumor recurrence and stage progression, but the study population had a low rate of BCG instillation (22.9%). Branchereau et al. [26] assessed the prognostic value of LVI in 108 patients with high grade T1 NMIBC, including 60 patients (57%) who received adjuvant BCG therapy after TURBT. They reported that LVI was not associated with tumor recurrence, stage progression, cancer-specific survival, or overall survival. In contrast, we clearly demonstrated that LVI status could independently predict recurrence and stage progression in T1 NMIBC patients receiving BCG therapy and that evaluation of LVI in tumor specimens could provide useful information planning the appropriate management strategy and patient counseling.

In the present series, the LVI status was only described for 54 patients (46.6%) in the original pathology reports. Among the remaining 62 patients (53.4%), a further 13 LVI-positive cases were found after review by our uro-pathologist. At our institution, some pathologists routinely evaluate LVI, while others do not mention the LVI status. One of the reasons for this difference might be that the predictive role of LVI status in TURBT specimens has not been established clinically, especially for T1 NMIBC. Berman et al. evaluated 2802 patients with muscle invasive bladder tumors treated by total cystectomy using the Ontario cancer registry dataset and found that pathology reports failed to address LVI status in 25%, with LVI reporting rates being significantly higher at the high volume centers for total cystectomy [30]. They concluded that assessment of LVI status in a standardized manner across all bladder specimens by pathologists is essential to improve the diagnostic and therapeutic strategies for this cancer. Our findings indicated that urologists and pathologists should share information concerning the prognostic significance of LVI, even in T1 NMIBC, emphasizing the importance of assessing and reporting LVI.

The present study had several limitations, including a small number of patients initially diagnosed with T1 bladder cancer and the long study period of 20 years, which was likely to have led to the low repeat TURBT rate (18.1%). Furthermore, we did not routinely perform maintenance BCG therapy in patients initially diagnosed with T1 bladder cancer and treatment after TURBT was not uniform. While 85 patients (73.3%) were treated with BCG, others received intravesical chemotherapy (13.8%) or no adjuvant therapy (12.9%). Therefore, we performed a subgroup analysis of the patients who received BCG therapy, which revealed that LVI status was an independent predictor of recurrence and stage progression in these patients. Since this study was retrospective, we cannot exclude the possibility of unknown biases, but we believe that consistent pathological data was obtained because the same uro-pathologist reviewed all of the specimens.

## Conclusions

The presence of LVI is a strong risk factor for tumor recurrence and stage progression in T1 NMIBC patients treated with BCG. This finding might assist in the development of appropriate management and counseling strategies for T1 NMIBC patients. Urologists and pathologists should be aware of the prognostic significance of LVI in T1 disease.

## Abbreviations

NMIBC: Non-muscle invasive bladder cancer
BCG: Bacillus Calmette-Guérin
CIS: Carcinoma in situ
TURBT: Transurethral resection of bladder tumor
LVI: Lymphovascular invasion
HR: Hazard ratio
